# Experience with Barium Chloride

**Published:** 1896-09

**Authors:** Jos. Plaskett

**Affiliations:** Nashville, Tenn.


### EXPERIENCE WITH BARIUM CHLORIDE.

By JOS. PLASKETT, D.V.S.,
NASHVILLE, TENN.

Having noticed in recent numbers of some of the veterinary journals numerous articles on the effects of barium chloride in the treatment of colic and similar affections in the horse, a few remarks regarding my experience with the drug may possibly be of some interest to the readers of your journal. After reading Prof. Dieckerhoff's exhaustive and interesting article on that subject and noting the uniformly happy results he obtained from the use of it, I came to the conclusion that it was the remedy the veterinarian had long been looking for. Pilocarpine and eserine have been selling at such prohibitive prices the last two years that their use, except in special cases, has been almost abandoned. When these drugs have been used and a bill is presented proportionate to the cost of the medicine used, the objection to it is generally so vigorous that some cheaper medication is usually tried in the next case.

My experience with barium chloride has not been so large as Prof. Dieckerhoff's, neither have the results I obtained been so uniformly successful. In fact, some of my results have been of the most serious nature; and although I have had some equally happy results, still I have come to the conclusion that the drug is not one that can be relied on with any degree of certainty. In some cases the exhibition of it has been followed by the good effects Prof. Dieckerhoff describes, and the speedy amelioration of the symptoms for which it was used; but in similar cases the administration of the same dose has produced effects which have been disastrous in the highest degree.

I shall endeavor to give as clearly and concisely as possible some of the results I have obtained from its use. When I first began using it I thought I would try the effect of it when given per orem in a capsule. The dose when exhibited in that manner is, according to Dieckerhoff, from two to three drachms. The first subject which presented itself for treatment was a gelding of medium size, which was brought to the infirmary one night suffering from colic. From the history I obtained, the horse had not been affected for more than half an hour, and on examination I found pulse and temperature normal and the animal not evincing many symptoms of pain. I administered at 6 P.M. 2½ drachms barium chloride in a capsule, and watched the animal for about half an hour. At that time he seemed to be entirely free from pain and was resting easily. I saw him at intervals until 11 o'clock, at which time he presented the following symptoms: Intestinal peristalsis much augmented, although no action from the bowels as yet; pulse 60; animal seemingly free from pain, but showed symptoms of depression, with rather an anxious expression of countenance. Thinking everything was going on smoothly, I left him for the night, but was called at 5 A.M. next morning, and found the horse in the following condition: Animal extended on its side; pulse 120 and scarcely perceptible; flanks and abdominal walls retracted to such a degree that it looked as if he had been eviscerated, whilst behind him was a large pool of liquid excreta, and numerous similar pools were scattered around the stall. The animal was in a state of complete collapse, and in spite of heroic doses of powerful stimulants died in an hour.

*Post-mortem.* The stomach and intestines were completely empty, but, beyond patches of congestion and extravasation of blood in the stomach and small intestine, I could detect nothing abnormal; after this experience I came to the conclusion that the dose I had given was too large, so in the treatment of the next few cases I gave doses of from half a drachm to a drachm and a half, with sometimes fair results and sometimes no results at all. As it always took from two to four hours to have the desired effect when given in this manner, I decided to try the intravenous method. Remembering my experience with my first case, I reduced the prescribed dose of 15 or 20 grains just one-half, and gave from 7 to 10 grains, according to size and condition of the horse. With this method the effects were much better, and I have obtained some excellent results as far as producing the physiological effects of the drug were concerned. In cases of acute indigestion accompanied by extreme tympany and distention of the intestines and constant eructations of gas from the stomach, I believe the judicious use of barium chloride intravenously, and the use of the trocar if necessary, will save a large percentage of cases that would otherwise die. But while the evacuations from the intestine have been prompt and copious, and the passage of flatus correspondingly so, I do not remember a case thus treated that has not been followed by some very unpleasant symptoms. Superpurgation or possibly just the physiological effect of the drug carried to excess has been one of these, and has been exceedingly difficult to control, but I have had no more fatalities from that cause. The exhibition of the drug seems to be followed by a general prostration of the entire system, in which the following symptoms are most prominent: In the course of an hour and a half or two hours after the injection, and usually about time the purgative effect is abating, the pulse will increase in frequency until it reaches from 80 to 110, and will become weak and small. The animal appears stupid and dull; will stand in one place with its head down and does not appear to notice anything, or to be cognizant of its surroundings. These symptoms gradually wear off, however, but the character and frequency of the pulse have given me considerable uneasiness on more than one occasion. I have yet to see in some 25 or 30 cases treated both by the intravenous method and per orem any symptoms of paralysis following, which has been noted by Dieckerhoff and others. The symptoms I have described followed the intravenous injection of from 6 to 10 grains, as 10 grains is the maximum dose I have given.

Before closing I wish to describe a case I treated in that manner about three weeks ago. The patient, a mare of medium size, had been at pasture for some time, and was observed to be showing symptoms of colic, which continued for about an hour and a half without improvement, when I was sent for. On examining the mare I found her in the following condition: Pulse 48; full and strong respirations, somewhat accelerated evidently from the pain she was suffering; abdomen tympanitic from accumulation of gas in the intestines. Borborygmus was present to some extent, and at intervals the mare would look around at her side, lie down and roll, and show other symptoms of abdominal pain. On examining mucous membrane of eye it was seen to be somewhat injected and showed one or two small petechial spots, as if the mare was suffering from a slight attack of influenza, which has been very prevalent here for the last six months. Diagnosis, acute indigestion. Taking a ten-grain powder of pulv. barium chloride, I shook out about one-quarter of it, and, putting the rest into my hypodermic syringe, I dissolved it in water and injected it very carefully into the jugular vein. I withdrew the needle, and turning around I replaced the needle and syringe in the case, and on looking up again was surprised to see the mare staggering. I seized her head, but she swayed and staggered around for about half a minute and then fell dead. During the time she was staggering she had an evacuation from the bowels, which was semifluid and quite copious. I had no opportunity of holding a post-mortem until twenty-four hours afterward, and as the weather was extremely hot decomposition had advanced to such an extent that it was rather unsatisfactory work.

*Post-mortem lesions.* The stomach was filled with masticated and partially digested grass, and also contained an enormous quantity of bots. The small intestines were likewise partially filled with ingesta, and the cæcum and floating colon contained large quantities of the same. I failed to discover any pathological lesions in these organs. The lungs were somewhat congested, and the pleura showed evidences of a previous attack of inflammation in the shape of numerous old adhesions. The heart appeared normal. Where the right kidney should have been there was a large mass of disintegrated and broken-down tissue, which was about twice the size of an ordinary kidney, and contained in the centre a considerable quantity of pus. The brain and spinal cord were not examined.

What the mare died from, and whether the barium chloride was the direct cause of death, I am not prepared to say. There was no possibility of air getting into the vein in sufficient quantity to produce any disturbance. The owner afterward told me that where the action from her bowels came in contact with the grass that it killed every blade it touched. In conclusion, it appears to me that barium chloride will have to be further investigated and experimented with before it can be brought into general use by the every-day practitioner. It is evident that it cannot be used indiscriminately, and that it can only be used with the greatest care and circumspection. It is possible that the drug I have been using is at fault, and that it contains impurities which cause it in some cases to have such disastrous effects. As for myself, after a fair trial of the effects of barium chloride, I believe that for the present I have contributed enough to the advancement of the profession in that respect, and shall retire temporarily from the field, leaving others to carry on the good work, only trusting that they may be more fortunate than I have been in determining the happy medium, not only in the size of the dose, but also in the effect it produces.

---

The firm of W. R. Jenkins will issue this fall an authorized translation of Professor Kobert's *Practical Toxicology*, which will be another valuable addition to the literature of the veterinarian.

Among other contributions to veterinary literature to issue from the press of the same house is a compend of *Veterinary Materia Medica and Therapeutics*, by Dr. A. C. Hassloch, late lecturer on these subjects at the New York College of Veterinary Surgeons.

*Bulletin No.* 12 *of the U. S. Department of Agriculture*, under the direction of the Bureau of Animal Industry, relating to tapeworms of poultry, is a very timely and important contribution looking to the better control of certain diseases which are so disastrous to our poultry interests, and from which the aggregate of losses annually reaches startling figures. It will occasion much interest wherever it is read and studied, and will lead to a much greater interest in the subject and result in measures to control and limit these serious losses. It is profusely illustrated, and exhibits a thorough study of the life-history and development of these parasites. Much credit is due to the chief of the department in directing this channel of work, and the earnest, thorough work of Drs. Stiles and Hassall is well displayed throughout the pages of this report.

